# Crystal structure of bis­{*S*-hexyl 3-[4-(di­methyl­amino)­benzyl­idene]di­thio­carbazato-κ^2^ *N* ^3^,*S*}copper(II)

**DOI:** 10.1107/S2056989015009342

**Published:** 2015-05-28

**Authors:** E. Zangrando, M. S. Begum, R. Miyatake, M. C. Sheikh, Md. M. Hossain

**Affiliations:** a Department of Chemical and Pharmaceutical Sciences, via Giorgieri 1, 34127 Trieste, Italy; b Department of Chemistry, Rajshahi University, Rajshahi-6205, Bangladesh; c Center for Environmental Conservation and Research Safety, University of Toyama, 3190 Gofuku, Toyama, 930-8555, Japan; d Department of Applied Chemistry, Faculty of Engineering, University of Toyama, 3190 Gofuku, Toyama 930-8555, Japan

**Keywords:** crystal structure, copper(II) complex, di­thio­carbazate ligand

## Abstract

In the title complex, [Cu(C_16_H_24_N_3_S_2_)_2_], the Cu^II^ atom is coordinated by two azomethine N and two thiol­ate S atoms of the chelating Schiff base ligands, resulting in a distorted square-planar coordination environment. The S—Cu—N chelating angle is of 84.41 (5)°. The Cu^II^ atom is located on a crystallographic inversion centre, leading to a *trans* configuration of the *N*,*S*-chelating ligands.

## Abstract 2

In the title complex, the Cu^II^ atom exhibits a square-planar coordination geometry and is located on a crystallographic inversion centre, leading to a *trans* configuration of the *N*,*S*-chelating ligands.

## Chemical context   

Bidentate Schiff bases of *S*-methyl or *S*-benzyl di­thio­carbaza­tes and their metal complexes have received considerable attention for their possible bioactivities (Chan *et al.*, 2008 ▸; How *et al.*, 2008 ▸; Ali *et al.*, 2002 ▸; Chew *et al.*, 2004 ▸; Crouse *et al.*, 2004 ▸). As part of our ongoing structural studies on these S-containing Schiff bases (Howlader *et al.*, 2015 ▸; Begum *et al.*, 2015 ▸), we report herein the structure of a copper(II) complex with the (di­methyl­amino­benzyl­idene)di­thio­carbazate ligand.
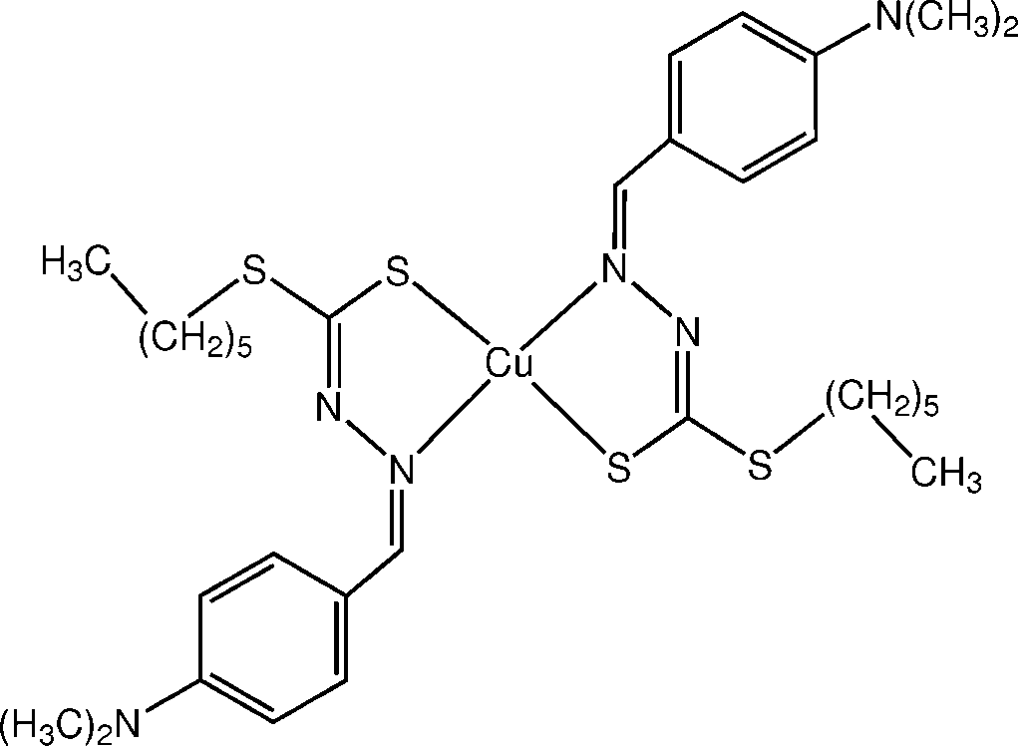


## Structural commentary   

**Figure 1:**
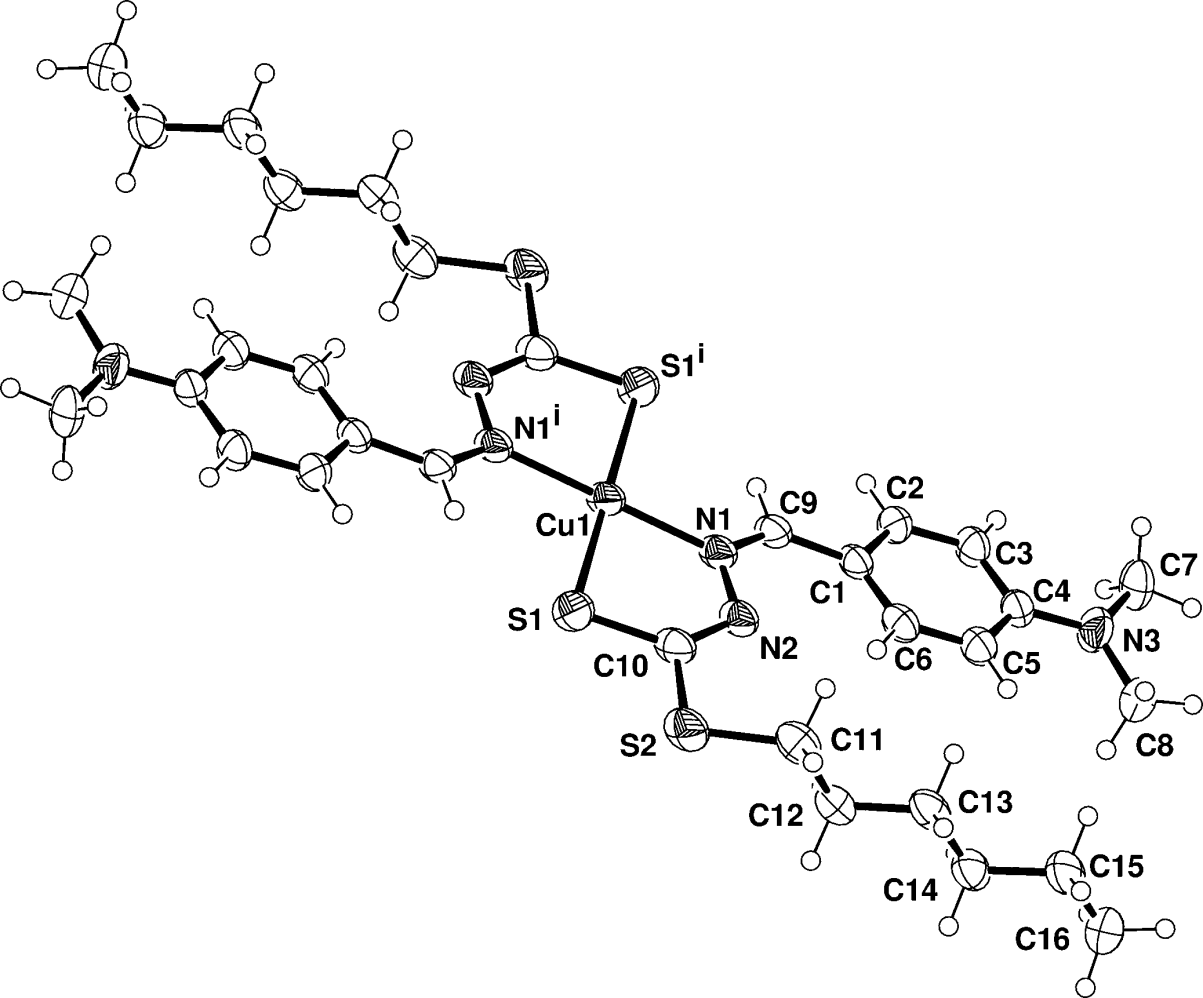
Drawing (ellipsoid probability at 50%) of the Cu*L*_2_ complex with atom labels of the crystallographic independent unit (primed atoms at −*x* + 2, −*y*, −*z* + 1).

In the crystal, the bis-chelated Cu^II^ complex resides on a crystallographic inversion centre and the two chelating Schiff bases, in their deprotonated imino thiol­ate form, coordinate the metal centre *via* the azomethine nitro­gen N1 and thiol­ate sulfur S1 atoms in a *trans*-planar configuration (Fig. 1 ▸). The Cu1—S and Cu1—N coordination bond lengths are of 2.2557 (6) and 2.0060 (14) Å, respectively, with an S1—Cu—N1 chelating angle of 84.41 (5)°. It is worth of note that copper(II) complexes with similar di­thio­carbazate ligands assume a distorted tetra­hedral configuration as well (Tarafder, *et al.*, 2008 ▸; Manan, *et al.*, 2011 ▸). In these derivatives the coordination distances are close comparable to those here reported. On the other hand the present Cu—S and Cu—N bond lengths are slightly longer with respect to those measured in the centrosymmetric complex with ligand bearing a benzyl group at the S atom [Cu—S = 2.165 (1), Cu—N = 1.929 (4) Å; Tian, *et al.*, 1998 ▸).

## Supra­molecular features   

**Figure 2:**
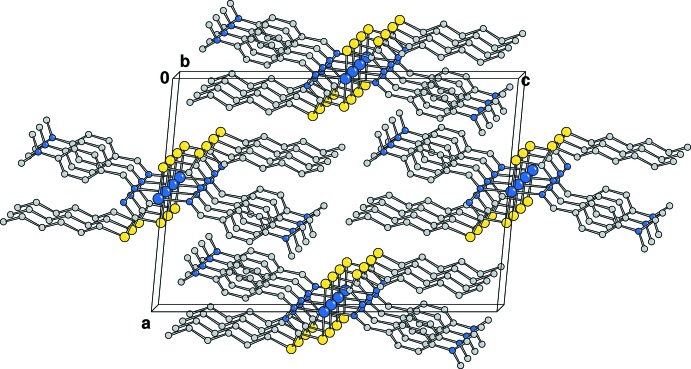
Crystal packing of the Cu*L*_2_ complex viewed down the *b* axis.

The crystal packing shows almost planar complexes piled along axis *b* with a stacking distance of 5.23947 (10) Å. (Fig. 2 ▸)

## Synthesis and crystallization   

A solution of Cu(CH_3_COO)_2_·H_2_O (0.10 g, 0.5 mmol, 15 mL methanol) was added to a solution of the *N*,*N*′-di­methyl­amino­benzaldehyde Schiff base of S-hexyl­dithio­carbazate (0.32 g, 1.0 mmol, 10 mL methanol). The resulting mixture was stirred at room temperature for seven hours. A dark reddish brown precipitate was formed, filtered off, washed with methanol and dried in vacuo over anhydrous CaCl_2_. Dark reddish brown single crystals of the compound, suitable for X-ray diffraction, were obtained by slow evaporation from a mixture of di­chloro­methane and aceto­nitrile (2:1), m.p. 437 K.

## Database survey   

The structure of the corresponding copper(II) complex with *N*,*N*′-di­methyl­amino­phenyl but having a benzyl group replac­ing the hexyl alkyl chain at S has been reported (Tian, *et al.*, 1998 ▸).

## Refinement   

Crystal data, data collection and structure refinement details are summarized in Table 1 ▸. All H atoms were fixed geom­etrically (C—H = 0.95–0.99 Å) and refined as riding, with *U*_iso_(H) = 1.2 *U*_eq_(C).

## Supplementary Material

Crystal structure: contains datablock(s) General, I. DOI: 10.1107/S2056989015009342/ff2136sup1.cif

Structure factors: contains datablock(s) I. DOI: 10.1107/S2056989015009342/ff2136Isup2.hkl

CCDC reference: 1057813

Additional supporting information:  crystallographic information; 3D view; checkCIF report

## Figures and Tables

**Table 1:** Experimental details

Crystal data	
Chemical formula	[Cu(C_16_H_24_N_3_S_2_)_2_]
*M*_r_	708.56
Crystal system, space group	Monoclinic, *P*2_1_/*n*
Temperature (K)	173
*a*, *b*, *c* ()	15.0457(4), 5.23947(10), 22.1944(5)
()	95.7007(7)
*V* (^3^)	1740.96(7)
*Z*	2
Radiation type	Mo *K*
(mm^1^)	0.90
Crystal size (mm)	0.24 0.17 0.05

Data collection	
Diffractometer	Rigaku R-AXIS RAPID
Absorption correction	Multi-scan (*ABSCOR*; Rigaku, 1995 ▸)
*T*_min_, *T*_max_	0.787, 0.956
No. of measured, independent and observed [*I* > 2(*I*)] reflections	16718, 3979, 3506
*R*_int_	0.023
(sin /)_max_ (^1^)	0.649

Refinement	
*R*[*F* ^2^ > 2(*F* ^2^)], *wR*(*F* ^2^), *S*	0.036, 0.102, 1.10
No. of reflections	3979
No. of parameters	199
H-atom treatment	H-atom parameters constrained
_max_, _min_ (e ^3^)	0.85, 0.49

Computer programs: *RAPID-AUTO* (Rigaku, 2001 ▸), *SIR92* (Altomare *et al.*, 1994 ▸), *SHELXL97* (Sheldrick, 2008 ▸) and *CrystalStructure* (Rigaku, 2010 ▸).

## supplementary crystallographic information

### Crystal data

**Table:**

[Cu(C_16_H_24_N_3_S_2_)_2_]	*F*(000) = 750.00
*M**_r_* = 708.56	*D*_x_ = 1.352 Mg m^−^^3^
Monoclinic, *P*2_1_/*n*	Mo *K*α radiation, λ = 0.71075 Å
Hall symbol: -P 2yn	Cell parameters from 14172 reflections
*a* = 15.0457 (4) Å	θ = 3.1–27.4°
*b* = 5.23947 (10) Å	µ = 0.90 mm^−^^1^
*c* = 22.1944 (5) Å	*T* = 173 K
β = 95.7007 (7)°	Platelet, red
*V* = 1740.96 (7) Å^3^	0.24 × 0.17 × 0.05 mm
*Z* = 2	

### Data collection

**Table:**

Rigaku R-AXIS RAPID diffractometer	3506 reflections with *I* > 2σ(*I*)
Detector resolution: 10.000 pixels mm^-1^	*R*_int_ = 0.023
ω scans	θ_max_ = 27.5°
Absorption correction: multi-scan (*ABSCOR*; Rigaku, 1995)	*h* = −19→19
*T*_min_ = 0.787, *T*_max_ = 0.956	*k* = −6→6
16718 measured reflections	*l* = −28→28
3979 independent reflections	

### Refinement

**Table:**

Refinement on *F*^2^	Secondary atom site location: difference Fourier map
*R*[*F*^2^ > 2σ(*F*^2^)] = 0.036	Hydrogen site location: inferred from neighbouring sites
*wR*(*F*^2^) = 0.102	H-atom parameters constrained
*S* = 1.10	*w* = 1/[σ^2^(*F*_o_^2^) + (0.0579*P*)^2^ + 0.429*P*] where *P* = (*F*_o_^2^ + 2*F*_c_^2^)/3
3979 reflections	(Δ/σ)_max_ = 0.002
199 parameters	Δρ_max_ = 0.85 e Å^−^^3^
0 restraints	Δρ_min_ = −0.49 e Å^−^^3^
Primary atom site location: structure-invariant direct methods	

### Special details

**Table:**

Geometry. ENTER SPECIAL DETAILS OF THE MOLECULAR GEOMETRY
Refinement. Refinement was performed using all reflections. The weighted *R*-factor (*wR*) and goodness of fit (*S*) are based on *F*^2^. *R*-factor (gt) are based on *F*. The threshold expression of *F*^2^ > 2.0 σ(*F*^2^) is used only for calculating *R*-factor (gt).

### Fractional atomic coordinates and isotropic or equivalent isotropic displacement parameters (Å^2^)

**Table:**

	*x*	*y*	*z*	*U*_iso_*/*U*_eq_	
Cu1	1.0000	0.0000	0.5000	0.04758 (12)	
S1	1.12679 (3)	0.22896 (13)	0.49888 (2)	0.06611 (19)	
S2	1.17728 (3)	0.56787 (10)	0.40439 (2)	0.05451 (15)	
N1	0.98526 (9)	0.0699 (3)	0.41070 (6)	0.0427 (4)	
N2	1.03819 (9)	0.2568 (3)	0.38625 (6)	0.0421 (3)	
N3	0.83204 (12)	−0.0665 (4)	0.11956 (7)	0.0572 (5)	
C1	0.91075 (10)	−0.0399 (3)	0.30708 (7)	0.0362 (4)	
C2	0.84924 (10)	−0.2217 (4)	0.28137 (7)	0.0392 (4)	
C3	0.82330 (11)	−0.2318 (3)	0.22036 (7)	0.0403 (4)	
C4	0.85831 (11)	−0.0592 (4)	0.18022 (7)	0.0388 (4)	
C5	0.92043 (11)	0.1218 (4)	0.20563 (8)	0.0429 (4)	
C6	0.94524 (10)	0.1320 (4)	0.26705 (8)	0.0405 (4)	
C7	0.76158 (14)	−0.2364 (4)	0.09510 (8)	0.0551 (5)	
C8	0.87505 (14)	0.0873 (5)	0.07706 (8)	0.0565 (5)	
C9	0.93057 (10)	−0.0546 (4)	0.37229 (7)	0.0407 (4)	
C10	1.10238 (11)	0.3347 (4)	0.42509 (8)	0.0452 (4)	
C11	1.12802 (12)	0.6732 (4)	0.33101 (9)	0.0496 (4)	
C12	1.15878 (11)	0.5323 (3)	0.27685 (9)	0.0439 (4)	
C13	1.11147 (11)	0.6348 (4)	0.21775 (9)	0.0459 (4)	
C14	1.13850 (13)	0.5022 (4)	0.16172 (9)	0.0487 (5)	
C15	1.08905 (13)	0.5981 (5)	0.10294 (10)	0.0565 (5)	
C16	1.11550 (17)	0.4573 (6)	0.04766 (10)	0.0715 (7)	
H1	0.8248	−0.3418	0.3072	0.0470*	
H2	0.7812	−0.3567	0.2050	0.0484*	
H3	0.9459	0.2399	0.1797	0.0515*	
H4	0.9866	0.2584	0.2826	0.0486*	
H5	0.7793	−0.4136	0.1039	0.0662*	
H6	0.7511	−0.2124	0.0512	0.0662*	
H7	0.7067	−0.1982	0.1137	0.0662*	
H8	0.8469	0.0556	0.0360	0.0678*	
H9	0.9385	0.0427	0.0792	0.0678*	
H10	0.8689	0.2682	0.0871	0.0678*	
H11	0.8968	−0.1807	0.3906	0.0488*	
H12	1.0623	0.6556	0.3298	0.0595*	
H13	1.1414	0.8569	0.3267	0.0595*	
H14	1.1459	0.3479	0.2804	0.0527*	
H15	1.2241	0.5531	0.2765	0.0527*	
H16	1.0462	0.6158	0.2189	0.0551*	
H17	1.1245	0.8193	0.2148	0.0551*	
H18	1.1275	0.3168	0.1654	0.0584*	
H19	1.2034	0.5264	0.1598	0.0584*	
H20	1.0240	0.5780	0.1050	0.0678*	
H21	1.1015	0.7823	0.0985	0.0678*	
H22	1.1790	0.4859	0.0438	0.0858*	
H23	1.0800	0.5210	0.0114	0.0858*	
H24	1.1046	0.2742	0.0522	0.0858*	

### Atomic displacement parameters (Å^2^)

**Table:**

	*U*^11^	*U*^22^	*U*^33^	*U*^12^	*U*^13^	*U*^23^
Cu1	0.02944 (16)	0.0819 (3)	0.03109 (16)	−0.01276 (13)	0.00126 (11)	−0.01776 (13)
S1	0.0422 (3)	0.1173 (5)	0.0373 (3)	−0.0323 (3)	−0.00378 (18)	−0.0104 (3)
S2	0.0418 (3)	0.0645 (3)	0.0554 (3)	−0.0158 (2)	−0.0040 (2)	−0.0138 (3)
N1	0.0291 (7)	0.0632 (9)	0.0353 (7)	−0.0061 (6)	0.0014 (6)	−0.0142 (7)
N2	0.0318 (7)	0.0544 (8)	0.0393 (7)	−0.0057 (6)	−0.0004 (6)	−0.0139 (6)
N3	0.0543 (10)	0.0814 (12)	0.0336 (8)	−0.0194 (9)	−0.0065 (7)	0.0048 (8)
C1	0.0267 (7)	0.0471 (9)	0.0337 (8)	0.0006 (6)	−0.0019 (6)	−0.0090 (6)
C2	0.0361 (8)	0.0464 (9)	0.0341 (8)	−0.0067 (7)	−0.0012 (6)	−0.0027 (7)
C3	0.0387 (8)	0.0443 (9)	0.0363 (8)	−0.0055 (7)	−0.0049 (7)	−0.0045 (7)
C4	0.0333 (8)	0.0477 (9)	0.0341 (8)	0.0025 (7)	−0.0024 (6)	−0.0016 (7)
C5	0.0360 (8)	0.0486 (9)	0.0430 (9)	−0.0039 (7)	−0.0016 (7)	0.0045 (7)
C6	0.0317 (8)	0.0439 (9)	0.0445 (9)	−0.0043 (6)	−0.0040 (7)	−0.0050 (7)
C7	0.0637 (12)	0.0624 (12)	0.0358 (9)	−0.0053 (9)	−0.0127 (8)	−0.0041 (8)
C8	0.0594 (12)	0.0718 (13)	0.0379 (9)	0.0012 (10)	0.0025 (8)	0.0085 (9)
C9	0.0279 (7)	0.0589 (10)	0.0350 (8)	−0.0053 (7)	0.0016 (6)	−0.0101 (7)
C10	0.0325 (8)	0.0612 (10)	0.0418 (9)	−0.0057 (7)	0.0025 (7)	−0.0180 (8)
C11	0.0399 (9)	0.0428 (9)	0.0647 (12)	−0.0006 (7)	−0.0017 (8)	−0.0078 (8)
C12	0.0339 (8)	0.0409 (8)	0.0559 (11)	0.0017 (6)	−0.0010 (7)	0.0008 (7)
C13	0.0355 (8)	0.0374 (8)	0.0636 (11)	0.0020 (7)	−0.0015 (8)	0.0082 (8)
C14	0.0400 (9)	0.0490 (10)	0.0561 (11)	0.0058 (7)	0.0000 (8)	0.0137 (8)
C15	0.0447 (10)	0.0612 (11)	0.0625 (12)	0.0047 (9)	−0.0002 (9)	0.0239 (10)
C16	0.0617 (14)	0.1008 (18)	0.0516 (12)	0.0101 (12)	0.0035 (10)	0.0275 (12)

### Geometric parameters (Å, º)

**Table:**

Cu1—S1	2.2557 (6)	C2—H1	0.950
Cu1—S1^i^	2.2557 (6)	C3—H2	0.950
Cu1—N1	2.0060 (14)	C5—H3	0.950
Cu1—N1^i^	2.0060 (14)	C6—H4	0.950
S1—C10	1.7333 (19)	C7—H5	0.980
S2—C10	1.7540 (19)	C7—H6	0.980
S2—C11	1.807 (2)	C7—H7	0.980
N1—N2	1.405 (2)	C8—H8	0.980
N1—C9	1.300 (2)	C8—H9	0.980
N2—C10	1.295 (2)	C8—H10	0.980
N3—C4	1.365 (3)	C9—H11	0.950
N3—C7	1.448 (3)	C11—H12	0.990
N3—C8	1.442 (3)	C11—H13	0.990
C1—C2	1.409 (3)	C12—H14	0.990
C1—C6	1.401 (3)	C12—H15	0.990
C1—C9	1.450 (3)	C13—H16	0.990
C2—C3	1.372 (3)	C13—H17	0.990
C3—C4	1.408 (3)	C14—H18	0.990
C4—C5	1.409 (3)	C14—H19	0.990
C5—C6	1.378 (3)	C15—H20	0.990
C11—C12	1.521 (3)	C15—H21	0.990
C12—C13	1.526 (3)	C16—H22	0.980
C13—C14	1.515 (3)	C16—H23	0.980
C14—C15	1.522 (3)	C16—H24	0.980
C15—C16	1.518 (4)		
			
S1—Cu1—S1^i^	180.00 (3)	N3—C7—H7	109.471
S1—Cu1—N1	84.41 (5)	H5—C7—H6	109.470
S1—Cu1—N1^i^	95.59 (5)	H5—C7—H7	109.466
S1^i^—Cu1—N1	95.59 (5)	H6—C7—H7	109.470
S1^i^—Cu1—N1^i^	84.41 (5)	N3—C8—H8	109.474
N1—Cu1—N1^i^	180.00 (9)	N3—C8—H9	109.468
Cu1—S1—C10	94.61 (6)	N3—C8—H10	109.474
C10—S2—C11	103.41 (9)	H8—C8—H9	109.477
Cu1—N1—N2	119.84 (10)	H8—C8—H10	109.470
Cu1—N1—C9	123.85 (13)	H9—C8—H10	109.464
N2—N1—C9	116.27 (14)	N1—C9—H11	113.512
N1—N2—C10	112.12 (14)	C1—C9—H11	113.515
C4—N3—C7	121.09 (17)	S2—C11—H12	108.342
C4—N3—C8	121.64 (17)	S2—C11—H13	108.340
C7—N3—C8	117.24 (15)	C12—C11—H12	108.348
C2—C1—C6	116.61 (14)	C12—C11—H13	108.348
C2—C1—C9	115.46 (15)	H12—C11—H13	107.429
C6—C1—C9	127.93 (15)	C11—C12—H14	109.470
C1—C2—C3	122.46 (16)	C11—C12—H15	109.469
C2—C3—C4	120.79 (15)	C13—C12—H14	109.463
N3—C4—C3	121.06 (16)	C13—C12—H15	109.473
N3—C4—C5	121.94 (17)	H14—C12—H15	108.061
C3—C4—C5	117.00 (15)	C12—C13—H16	108.791
C4—C5—C6	121.71 (16)	C12—C13—H17	108.792
C1—C6—C5	121.43 (15)	C14—C13—H16	108.801
N1—C9—C1	132.97 (16)	C14—C13—H17	108.796
S1—C10—S2	112.88 (10)	H16—C13—H17	107.674
S1—C10—N2	127.03 (15)	C13—C14—H18	108.802
S2—C10—N2	120.09 (14)	C13—C14—H19	108.799
S2—C11—C12	115.75 (13)	C15—C14—H18	108.789
C11—C12—C13	110.86 (14)	C15—C14—H19	108.785
C12—C13—C14	113.82 (15)	H18—C14—H19	107.662
C13—C14—C15	113.83 (16)	C14—C15—H20	109.040
C14—C15—C16	112.79 (18)	C14—C15—H21	109.036
C1—C2—H1	118.769	C16—C15—H20	109.026
C3—C2—H1	118.774	C16—C15—H21	109.020
C2—C3—H2	119.601	H20—C15—H21	107.805
C4—C3—H2	119.605	C15—C16—H22	109.477
C4—C5—H3	119.145	C15—C16—H23	109.474
C6—C5—H3	119.143	C15—C16—H24	109.466
C1—C6—H4	119.289	H22—C16—H23	109.466
C5—C6—H4	119.283	H22—C16—H24	109.475
N3—C7—H5	109.479	H23—C16—H24	109.470
N3—C7—H6	109.472		

Symmetry code: (i) −*x*+2, −*y*, −*z*+1.
